# Antenatal services for pregnant teenagers in Mbarara Municipality, Southwestern Uganda: health workers and community leaders’ views

**DOI:** 10.1186/s12884-015-0772-0

**Published:** 2015-12-23

**Authors:** Godfrey Zari Rukundo, Catherine Abaasa, Peace Byamukama Natukunda, Bob Harold Ashabahebwa, Dominic Allain

**Affiliations:** Department of Psychiatry, Mbarara University of Science and Technology, P. O. Box 1410, Mbarara, Uganda; Kampala International University, Western Campus, Bushenyi, Uganda; Uganda Christian University, Kampala, Uganda; Insurance Company of East Africa, Mbarara, Uganda; Department of Paediatrics, University of Alberta, Calgary, AB Canada

**Keywords:** Teenagers, Pregnancy, Antenatal services, Community leaders, Health workers

## Abstract

**Background:**

Globally, about 11 % of all annual births involve adolescents aged 15–19 years. Uganda has one of the highest teenage pregnancy rates in Sub-Saharan Africa. This study assessed stakeholders’ views concerning factors affecting availability, accessibility and utilization of teenager friendly antenatal services in Mbarara Municipality, southwestern Uganda.

**Method:**

This was a cross-sectional descriptive study utilizing Key Informant Interviews (KIIs). It was conducted in three divisions of Mbarara Municipality. The KIIs were held six Village Health Team (VHT) members, three gynecologists, six midwives, three Community leaders (LC 3 Secretaries for women affairs), one police officer from the Family and Child protection unit at Mbarara Police and three Traditional Birth Attendants (TBAs). Data analysis was done manually by identifying emergent themes which were later coded and organized into concepts which were later developed into explanations.

**Results:**

Reproductive health stakeholders generally considered teenage pregnancy to be among the high risk pregnancies that need to be handled with care. In addition, the reproductive health workers described their experience with teenagers as challenging due to their limited skills when it comes to addressing adolescent-specific needs. Adolescent-friendly services were defined as those that could provide privacy, enough time and patience when dealing with teenagers. With this description, there were no teenager-friendly antenatal services in Mbarara municipality at the time of the study. There is need for proactive steps to establish these services if the needs of this subgroup are to be met.

**Conclusion:**

There are no teenager friendly antenatal services in Mbarara municipality and few teenagers access and utilise the available general antenatal services. There is need for specialized training for health workers who deal with pregnant teens in Mbarara Municipality in order for them to provide teenager friendly services.

## Background

Globally, about 95 % of births among women aged 15–19 years occur in low-and middle-income countries [1, 2]. The proportion of births that takes place during adolescence represents more than 50 % of births in Sub-Saharan Africa. Regrettably, many health problems are particularly associated with adolescent pregnancies including maternal mortality [3]. In Uganda, where maternal mortality stands at 438 deaths per 100,000 live births, about 24 % of mothers who die are teenagers. Availability, accessibility and utilization of antenatal services are essential for reproductive health and for the development of a nation [4–7]. At least four antenatal visits for every pregnant woman including teenagers are recommended [8]. However, according to the Uganda Demographic Health survey of 2006, the recommended general antenatal care attendance (4+ visits) stands at 48 %, and about 25 % of teenagers become pregnant annually. In addition to increased risks of mortality, teenagers are also at increased risk of other poor maternal and infant outcomes [9, 10] and carry greater burdens of sexually transmitted infections, violence, poor social support, missed opportunities and psychological distress [2, 9, 11–16]. Most reproductive health services are designed to meet the antenatal needs of adult mothers [15] but may not be appropriate for teenagers [17]. Yet, teenagers represent a vulnerable group that needs to be given special consideration. For this to happen, stakeholders in reproductive health services need to be involved in the planning and implementation of teenager friendly services [18, 19]. Hence teenage pregnancy, its causes and consequences need further investigation. Various stakeholders need to be involved in the studies so as to come up with uniform interventions. This study assessed stakeholders’ views concerning availability, accessibility and utilization of teenager friendly antenatal services in three divisions of Mbarara Municipality southwestern Uganda.

## Methods

This was a cross-sectional descriptive study utilizing qualitative data collection and analysis methods. It was conducted in three divisions of Mbarara Municipality: Kamukuzi, Nyamitanga and Kakoba, in southwestern Uganda. At the time of data collection, the municipality was made up of these three divisions. Recently, three other divisions (Nyakayojo, Biharwe and Kakiika) were added to the Municipality in preparation for city status. Mbarara municipality is the biggest business town in the western region, approximately 270 kilometers from the city of Kampala. It is surrounded by Kiruhura and Lyantonde districts in the north, Ntungamo district in the south, Sheema and Ibanda districts form the western boarder and on the east side is the Isingiro district.

The Key Informant Interviews were selected from three divisions basing on their involvement in adolescent reproductive health. These included six VHT members, three gynaecologists, six midwives, three Community leaders (LC 3 Secretaries for women affairs), one police officer from the Family and Child protection unit at Mbarara Police and three Traditional Birth Attendants (TBAs). This data was collected over a period of six months.

The KIIs in this study were purposively selected because of their position, involvement, knowledge or experience in reproductive health services and the plight of adolescents in Mbarara municipality. The gynaecologiststs and midwives routinely attend to pregnant women requiring antenatal care. The VHTs and TBAs are the usual points of first contact when members of the community have health needs. The local leaders, the family protection unit of the police and the probation office usually get involved when an adolescent becomes pregnant. The study participants were interviewed at their places of work. They were initially contacted for appointment and later interviewed at their convenience. The interviews took different periods depending on the amount of information the participant was ready to provide. On average, each interview was 30–45 min.

The study was approved by the Mbarara University of Science and Technology Research and Ethics Committee of (MUST-REC). Permission was also sought from Mbarara municipality administration, Health Centre In-charges before data collection. Written informed consent/assent from individual participants was obtained before they participated in the study. Specific consent was sought from participants whose audio information was recorded. Participants were informed that their privacy and confidentiality would be respected and that there was no potential harm associated with participating in the study. It was made clear to the participants that participation in the study was voluntary and that they were free to opt out of the study at any time without any negative consequences.

Data was collected by three of the authors with the assistance of three trained research assistants. During interviews, documentation of data was performed by two research team members writing notes. The notes were then compared and later combined to make one set of data. Data analysis was done manually by identifying emergent themes which were later coded and organized into concepts, which were later developed into tentative explanations. This further gave the authors opportunity to familiarize themselves with the data. Diagramming (assessing the linkages and meanings) the relationship among concepts was done to demonstrate how one concept influenced another so as to create generalization, trends and conclusions.

## Results and discussion

According to the study participants, there were no teenager friendly antenatal services in Mbarara municipality. The teenagers requiring antenatal care are expected to attend the adult oriented services. The attendance of antenatal services by teenagers is low at all the health units in the municipality. Despite the unavailability of antenatal services specifically for teenager, the gynecologists and midwives were aware of what could constitute teenager friendly antenatal services.

### Health workers views on what would make an adolescent friendly service

Health workers viewed a teenager-friendly service as one that could provide privacy and sufficient time and patience when dealing with teenagers. They also described that a friendly service would be offered by health workers with specific training in teenage pregnancy and with knowledge of how to allocate specific time to teenagers. They also described health education and specific space for the teenagers as key components of a teenager friendly service. In addition, a good relationship between the patient and the midwives should characterise the service.

*‘A teenager friendly service should contain health education, HIV testing and counseling, family planning, treatment of STIs and offering Antiretroviral Therapy to the HIV positive youths’ (Gynecologist)*

*‘A service with privacy; teenage mothers need enough time, patience, specific training in teenage pregnancy for them to know how to be allocated specific time. They need health education, specific space for them and enough trained personnel with different skills” (Midwife)*

According to the above description, such services were not available in Mbarara Municipality at the time of the study. However, pregnant teenagers are viewed as a special group requiring special attention.

### Health workers views on Teenagers as a special group requiring attention

The Reproductive health workers in Mbarara Municipality considered teenage pregnancies to be among the high risk pregnancies they handled. However, they did not consider the women’s young age as the main factor. Instead, they considered possible challenges that may be associated with young age like contracted pelvis.

*‘High risk pregnancies are those in which mothers have medical complications. High risk pregnancies may include teenage pregnancies but being teenagers alone does not necessarily mean that the mothers should be considered as special group that needs special attention’. (Gynecologist)*

However, the teenagers were viewed as a group for which special skills were required when managing. The health workers reported that they did not have sufficient skills required while handling pregnant teenagers. Being able to obtain information from the reluctant teenagers was a skill considered essential in teenager antenatal care services.

*‘Working with pregnant teenagers is challenging; some don’t know they are pregnant, they deny and need a lot of time to understand.’ (Midwife)*

### Health workers views on factors affecting accessibility and utilisation of Antenatal Care services by adolescents

The reproductive health workers reported that teenagers were not utilizing the available reproductive health services as expected. A number of factors were raised by the midwives involved in the study. For example, teenagers were considered as impatient; they often require a quick service in which they do not have to wait for long. This is usually not possible in the general antenatal services where there are many mothers with different concerns. In addition, the teenagers would prefer to be on their own, not mixing them with adults.

*‘Teenagers don’t want to sit with adults, they fear adults, and there is inadequate privacy in the general antenatal services. Teenagers are afraid to go to the clinics for fear of either being seen or abused by older women and health workers’ (Midwife)*

*‘Teenagers may not be aware of the available antenatal services and lack support because most of them are dependants.’ (Midwife)*

In addition, most of the girls between 13 and 17 years get pregnant unwillingly and sometimes they are infected with HIV/AIDS and other STIs. This further complicates antenatal care seeking. They often rejected by the men responsible for pregnancy and stay with their parents or grandparents but with limited social and financial support.

When the teenagers gain courage or support to seek antenatal care, they often find reproductive health services which are not prepared for them. The gynaecologists and midwives find their experience with teenagers as challenging due to limited skills in adolescent reproductive health.

*‘It has not been easy when trying to talk to the adolescents because some girls come when they are complaining of headache and stomachache even when they know that they are pregnant and at the end they tell the truth but not willingly. It is so challenging in that when young girls come, they come with the aim of aborting even when they have been counseled.’ (Mid-wife)*

### Availability of support for pregnant teenagers in the community

The main support for pregnant teenagers in the community was described by Village Health Team (VHT) members as provision of information and interventions to improve social relations with family members *(VHT member).*

*‘When we get some of the pregnant teenagers, we sit with them, counsel them, and talk to their parents not to harass them but to support them.’ ‘We also tell them to attend ANC in hospitals’. (VHT member)*

When a teenager becomes pregnant, the community members find themselves ill prepared to support the teenagers. The communities in Mbarara Municipality lack specific interventions for pregnant teenagers. However, the teenagers benefit from the advice and support from members of the community.

*‘As VHTs, we were given first aid boxes but there is nothing in them; they are just kept.’ ‘There is no material support given to the pregnant teenagers. We offer some little emotional support and help in looking for the men responsible for the pregnancy.’ (VHT member)*

*‘We try to sensitize the communities about antenatal care services through VHTs, Local Councils; it is every body’s responsibility since it is government policy’ (LC III Secretary for women)*

Teenagers may not be able to access and utilize the available antenatal care services due to lack sufficient social support. As a result the teenagers tend to try many things to survive. This further puts their lives and unborn babies at increased risk.

‘*I once came across a teenager who confessed that she locked a child in the house to go to the memory pub do prostitution to earn a living. When I asked that doesn’t the child cry? She said, I give the child piriton tablets to sleep’. (LC III Secretary for women)*

### Discussion

This study assessed stakeholders’ views concerning availability, accessibility and utilization of teenager friendly antenatal services in three divisions of Mbarara Municipality southwestern Uganda. The study findings revealed that although it was necessary to have teenager friendly antenatal services, there were no antenatal services specifically targeting the needs of pregnant teenagers in the three divisions. This is not in keeping with the sustainable development goals in which maternal and child health is a major focus. Having no teenager friendly ANC services seems to be a challenge of prioritization. If Uganda was to focus on the needs of the population, children and adolescents who contribute more than 50 % of the population would have better services. The young people are the future of the country.

The reproductive health workers in the current study reported teenage pregnancy to be in the category of high risk pregnancies. Pregnant teenagers face multiple challenges and may not be able to access and utilize the available antenatal services. The fact that teenage pregnancy is associated with poor health seeking behavior has also been reported in previous studies [20]. In addition, teenage pregnancy can result in serious, long-term negative health effects including unsafe abortion, missed opportunities, school dropout and maternal mortality [1, 21, 22]. It is important to note that most of the teenage pregnancies are unintended [23]. Other issues like missed opportunities while pregnant and nursing the baby and lack of skills for parenthood were not considered being strong enough to make teenage pregnancies high risk pregnancies.

According to this study, providing antenatal care to teenagers is quite challenging for reproductive health workers. This could be due to lack of specific training in how to deal with adolescents. This finding concurs with other studies which indicated that teenagers tend to stay away from the service or default from clinic attendance [24]. The environment in which the teenagers are expected to seek service is not conducive. They feel uncomfortable being in the same clinics with the older mothers/women. The age difference between themselves and other women attending the clinic may make them perceive themselves as inferior and as being treated as such at the clinic. This may be embarrassing to them [24].

Vague symptoms, unreliable menstrual history, and adolescent reluctance to disclose sexual activity present challenges to early diagnosis. When pregnancy is suspected, clinicians need skills for accurate diagnosis, conducting comprehensive assessments, and providing options counseling. These skills seem to be missing among the reproductive health workers in the Mbarara municipality, but also likely in other areas of Sub-Saharan Africa as well.

This study is limited by the fact that it was conducted among few stakeholders in teenager reproductive health. There are other stakeholders like religious leaders, community elders and cultural leaders who were not interviewed. Due to the study design, the findings of this study cannot be generalized to the entire population in the municipality.

## Conclusions

According to stakeholders for adolescent reproductive health, there are no teenager friendly antenatal services in Mbarara municipality. Few teenagers access and utilise the available general antenatal services.

### Recommendations

Antenatal services specifically targeting teenagers are necessary. There is need to create more specialized training for health care workers who deal with pregnant teens in Mbarara Municipality. There is a need for further research in this area, for example, implementing a new strategy to improve accessibility and utilization of antenatal services by adolescents. This interventional research could be done by establishing antenatal services specifically for teenagers and evaluating its successes and needs for improvement.
